# Application and comparison of scoring indices to predict outcomes in patients with healthcare-associated pneumonia

**DOI:** 10.1186/cc9979

**Published:** 2011-01-19

**Authors:** Wen-Feng Fang, Kuang-Yao Yang, Chieh-Liang Wu, Chong-Jen Yu, Chang-Wen Chen, Chih-Yen Tu, Meng-Chih Lin

**Affiliations:** 1 Division of Pulmonary and Critical Care Medicine and Department of Respiratory Therapy, Kaohsiung Chang Gung Memorial Hospital, Chang Gung University College of Medicine, Ta-Pei Road, Kaohsiung 833, Taiwan; 2 Department of Respiratory Care, Chang Gung Institute of Technology, Chia-pu Road, Chiayi 813, Taiwan; 3 Chest Department, Taipei Veterans General Hospital, Shipai Road, Taipei 112, and Institute of Clinical Medicine, School of Medicine, National Yang-Ming University, Linong Street, Taipei 112, Taiwan; 4 Division of Critical Care & Respiratory Therapy, Department of Internal Medicine, Taichung Veterans General Hospital, Chung-Kang Road, Taichung 407, Taiwan; 5 Department of Internal Medicine, National Taiwan University Hospital, RenAi Road, Taipei 106, Taiwan; 6 Medical Intensive Care Unit, Department of Internal Medicine, National Cheng-Kung University Hospital, Sheng Li Road, Tainan 704, Taiwan; 7 Division of Pulmonary and Critical Care Medicine, Department of Internal Medicine, China Medical University Hospital, Yuh-Der Road, Taichung 404, Taiwan; 8 Division of Pulmonary and Critical Care Medicine, Xiamen Chang Gung Hospital, Xiafei Road, Xiamen 361000, China

## Abstract

**Introduction:**

Healthcare-associated pneumonia (HCAP) is a relatively new category of pneumonia. It refers to infections that occur prior to hospital admission in patients with specific risk factors following contact or exposure to a healthcare environment. There is currently no scoring index to predict the outcomes of HCAP patients. We applied and compared different community acquired pneumonia (CAP) scoring indices to predict 30-day mortality and 3-day and 14-day intensive care unit (ICU) admission in patients with HCAP.

**Methods:**

We conducted a retrospective cohort study based on an inpatient database from six medical centers, recruiting a total of 444 patients with HCAP between 1 January 2007 and 31 December 2007. Pneumonia severity scoring indices including PSI (pneumonia severity index), CURB 65 (confusion, urea, respiratory rate, blood pressure, age 65), IDSA/ATS (Infectious Diseases Society of America/American Thoracic Society), modified ATS rule, SCAP (severe community acquired pneumonia), SMART-COP (systolic blood pressure, multilobar involvement, albumin, respiratory rate, tachycardia, confusion, oxygenation, pH), SMRT-CO (systolic blood pressure, multilobar involvement, respiratory rate, tachycardia, confusion, oxygenation), and SOAR (systolic blood pressure, oxygenation, age, respiratory rate) were calculated for each patient. Patient characteristics, co-morbidities, pneumonia pathogen culture results, length of hospital stay (LOS), and length of ICU stay were also recorded.

**Results:**

PSI (>90) has the highest sensitivity in predicting mortality, followed by CURB-65 (≥2) and SCAP (>9) (SCAP score (area under the curve (AUC): 0.71), PSI (AUC: 0.70) and CURB-65 (AUC: 0.66)). Compared to PSI, modified ATS, IDSA/ATS, SCAP, and SMART-COP were easy to calculate. For predicting ICU admission (Day 3 and Day 14), modified ATS (AUC: 0.84, 0.82), SMART-COP (AUC: 0.84, 0.82), SCAP (AUC: 0.82, 0.80) and IDSA/ATS (AUC: 0.80, 0.79) performed better (statistically significant difference) than PSI, CURB-65, SOAR and SMRT-CO.

**Conclusions:**

The utility of the scoring indices for risk assessment in patients with healthcare-associated pneumonia shows that the scoring indices originally designed for CAP can be applied to HCAP.

## Introduction

Healthcare-associated pneumonia (HCAP), a relatively new category of pneumonia, refers to infections that occur prior to hospital admission in patients with contact or exposure to a healthcare environment [1]. Compared to community-acquired pneumonia (CAP), HCAP is a distinct type of pneumonia with unique microbiological and epidemiological characteristics and outcomes [2-6].

In the current era of rising healthcare costs, the decision to hospitalize adults with CAP has received considerable attention and many pneumonia severity prediction rules have been designed to stratify patients with CAP into risk groups [7,8]. Severity assessment is not only the key to deciding the site of care but also in guiding both general management and antibiotic treatment. Of the prominent tools for this purpose are the Pneumonia Severity Index (PSI) developed by Fine and colleagues [9] and the CURB (confusion, urea, respiratory rate, blood pressure) score proposed by the British Thoracic Society, and Infectious Diseases Society of America/American Thoracic Society Consensus Guidelines on the Management of Community-Acquired Pneumonia in Adults [10]. Other clinical prediction rules for severe community-acquired pneumonia, like the severe community acquired pneumonia (SCAP) score were also developed, and were seemingly better at identifying severe CAP. The SCAP is validated to predict 30-day mortality among two cohorts of consecutive adult patients with CAP and identifies more patients as low risk for potential outpatient care [11]. The need for ICU care was better identified with the SOAR (systolic blood pressure, oxygenation, age, respiratory rate) model compared to the other scoring rules (CURB (confusion, urea, respiratory rate, blood pressure), CURB-65 (confusion, urea, respiratory rate, blood pressure, age 65), CRB-65 (confusion, respiratory rate, blood pressure, age 65)) in patients with nursing home acquired pneumonia [12], a subgroup of HCAP.

Each scoring system has its strengths and weaknesses. As demonstrated by the studies on heterogeneous populations, validation studies of algorithms for HCAP therapy will be difficult [13]. It would be very helpful if we can apply the existing scoring systems to HCAP. However, to the best of our knowledge, none of these prediction rules has been validated in patients hospitalized with HCAP. Therefore, we sought to compare the performance of the current scoring indices to predict mortality and ICU admission in patients with HCAP.

## Materials and methods

### Setting and study design

This multi-center study was conducted at six medical centers in Taiwan (Taipei Veterans General Hospital, National Taiwan University Hospital, Taichung Veterans General Hospital, China Medical University Hospital, National Cheng Kung University Hospital, and Kaohsiung Chang Gung Memorial Hospital). All adult patients presenting to one of the study hospitals with pneumonia who were discharged between 1 January 2007 and 31 December 2007 were reviewed. According to the 2005 IDSA/ATS (Infectious Diseases Society of America/American Thoracic Society) guidelines [14], a patient with HCAP is defined as one having pneumonia and any of the following historical features: (1) hospitalization for two or more days in an acute care facility within 90 days of infection, (2) being a resident of a nursing home or long-term care facility, (3) attending a hospital or hemodialysis clinic, (4) having received intravenous antibiotic, chemotherapy, or wound care within 30 days of infection. The patients were excluded if they had any one of the following conditions: (1) they were younger than 18 years old; (2) their pneumonia developed two days after admission or within 14 days after discharge; (3) they had lung cancer with obstructive pneumonia; (4) they were HIV positive with a CD4+ <200; (5) there were inadequate data for scoring. A total of 551 HCAP patients were recruited and 444 patients with adequate data (with all variables for calculating all scoring indices we compared available at admission) were studied. The study was approved by the institutional review board of each medical center and informed consent was waived.

### Microbiology evaluation

The specimens obtained within 72 h of admission were eligible for etiologic evaluation, including sputum, tracheal aspirate, bronchoalveolar lavage fluid, pleural effusion, blood, and urine for Legionellae antigen test or *Streptococcus pneumoniae *antigen test. The HCAP pathogens were defined according to the principles proposed by Lauderdale *et al*. [15].

In brief, etiology was determined based on laboratory data from blood and sputum cultures plus serology from paired serum and urine antigen detection tests. Blood cultures were accepted if the same microorganism was identified in a respiratory specimen and no other source for the positive blood culture could be identified. If the patients received bronchoscopic study, the definite organisms were confirmed by quantitative bacterial cultures BAL (bronchoalveolar lavage) >10^4^/cfu or PSB (protected sheath brushing) >10^3^/cfu. The probable pathogen was the organism isolated as a predominant organism from sputum or endotracheal aspirate.

### Definition of co-morbidities

The co-morbidities were defined according to the definition in the study by Fine *et al. *[9], including neoplastic disease, liver disease, congestive heart failure, cerebrovascular disease, and renal disease.

### Outcomes

The primary outcomes include 30-day all-cause mortality and ICU admission after 3 days and 14 days. The lengths of both the ICU and hospital stay were also determined.

### Scoring indices

**The modified ATS rule **was met if at least two of three minor criteria assessed at admission (systolic blood pressure <90 mmHg, multilobar (>2 lobes) involvement, PaO_2_/FiO_2 _<250), or one of two major criteria assessed at admission or during follow-up (requirement for mechanical ventilation or septic shock) were present [16,17].

**IDSA/ATS **refers to the Infectious Diseases Society of America/American Thoracic Society Consensus Guidelines on the Management of Community-Acquired Pneumonia in Adults [10]. In addition to the two major criteria (need for mechanical ventilation and septic shock), an expanded set of minor criteria (respiratory rate ≥30 breaths/minute; arterial oxygen pressure/fraction of inspired oxygen (PaO2/FiO2) ratio ≤250; multilobar infiltrates; confusion; blood urea nitrogen level ≥20 mg/dL; leukopenia resulting from infection; thrombocytopenia; hypothermia; or hypotension requiring aggressive fluid resuscitation) is proposed. The presence of at least three of these criteria suggests the need for ICU care.

**SOAR **comprises systolic blood pressure, oxygenation, age, and respiratory rate [18]. We then defined severe pneumonia as the presence of two or more out of the four criteria. A score of 1 was given for the presence of each of the following (dichotomized variables): systolic BP <90 mmHg; PaO2:FiO2 <250; age ≥65 years; and RR ≥30/minute.

**SCAP **was proposed by Espana [19]. The evaluation of SCAP is based on the presence of one major criterion (PS) or two or more minor criteria (CURXO80). P = arterial pH <7.3; S = systolic pressure <90 mmHg; C = confusion; U = blood urea nitrogen >30 mg/dL; R = respiratory rate >30/minute; X = X-ray multilobar bilateral; O = PaO_2 _<54 or PaO_2_/FiO_2 _<250 mmHg; and 80 = Age ≥80 years.

**SMART-COP **(systolic blood pressure, multilobar involvement, albumin respiratory rate, tachycardia, confusion, oxygenation, pH) scores were calculated as presented by Charles [20], and consisted of systolic blood pressure (<90 mmHg, two points); multilobar chest radiography involvement (one point); low albumin level (<3.5 g/dL, one point); high respiratory rate (≤50 years: ≥25 br/minute, >50 years: ≥30 br/minute; one point); tachycardia (≥125 bpm; one point); confusion (new onset; one point); poor oxygenation (≤50 years: PaO_2 _<70 mmHg or O_2 _saturation ≤93%, >50 years: PaO_2 _<60 mmHg or O_2 _saturation ≤90%; two points); and low arterial pH (<7.35; two points).

**SMRT-CO **(Simplified SMART-COP was designed for use by primary care physicians, and it excludes the results for albumin, arterial pH, and PaO_2 _[20]).

**CURB-65 score **is a six-point score, with one point for each of: confusion; urea >7 mmol/l; respiratory rate ≥30/minute; low systolic (<90 mmHg) or diastolic (≤60 mmHg) blood pressure; and age ≥65 years [21].

The pneumonia severity index (PSI) was calculated as presented in the study by Fine *et al. *[9], and is comprised of the following variables: age, gender, co-morbidity, and vital sign abnormalities, together with several laboratory, blood gas, and radiographic parameters. The PSI results in a five-class point scoring system reflecting the increasing risk of mortality.

### Statistical analysis

Categorical variables were analyzed using a chi-square test or Fisher's exact test where appropriate, and continuous variables were compared using Student's *t*-test or the Mann-Whitney U test. The discriminatory power of each scoring index was measured by receiver operating characteristic (ROC) curves. The areas under the ROC curve (AUC) was calculated to give an estimate of the overall accuracy of each scoring index in predicting different patient outcomes (3-day ICU admission, 14-day ICU admission and 30-day mortality). An area of 0.50 implies that the scoring index is no better than chance, whereas an area of 1 implies perfect accuracy. Sensitivity, specificity, positive predictive value (PPV), and negative predictive value (NPV) were also calculated as well with their 95% confidence intervals for all the scoring indices. The Hanley-McNeil test was used for testing the statistical significance of the difference between the two AUC figures. All tests were two-tailed, and *P-*value <0.05 was considered to be statistically significant. All statistical analyses were performed using the SPSS 14.0 software (SPSS Inc., Chicago, IL, USA) and the MedCalc 9.6.2.0 package (MedCalc Software, Mariakerke, Belgium).

## Results

### Enrolled background

A total of 444 patients met the inclusion criteria for HCAP. Among these patients, there were 40 (9%) patients receiving regular hemodialysis, peritoneal dialysis, or infusion therapy. The enrolled patient backgrounds are provided in Table 1. The all-cause mortality rate at 30 days was 20.9%, and the 3-day ICU admission and 14-day ICU admission rates were 25% and 29.1%, respectively.

**Table 1:** Background of patients with healthcare-associated pneumonia

			3-day		14-day		30-day	
		All	Non-ICU	ICU	Non-ICU	ICU	Survivors	Non-Survivors
		*N *= 444	*N *= 333	*N *= 111	*N *= 315	*N *= 129	*N *= 351	*N *= 93
I.*	Regular hemodialysis, peritoneal dialysis or infusion therapy	40 (9.0)	27 (8.1)	13 (11.7)	26 (8.3)	14 (10.9)	38 (10.8)	2 (2.2)
II.^#^	Chemotherapy in out-patient clinics within 90 days	92 (20.7)	74 (22.2)	18 (16.2)	70 (22.2)	22 (17.1)	60 (17.1)	32 (34.4)
III.^†^	Hospitalization for ≥2 days within 90 days before the onset of pneumonia	199 (44.8)	150 (45.0)	49 (44.1)	141 (44.8)	58 (45.0)	155 (44.2)	44 (47.3)
IV.	Residents in a nursing home or long-term care institute	113 (25.5)	82 (24.6)	31 (27.9)	78 (24.8)	35 (27.1)	98 (27.9)	15 (16.1)
			*P *= 0.388		*P *= 0.558		*P *< 0.001	

*The patients were classified into I if their enrolled background included I and the others (II, III or IV)

#The patients were classified into II if their enrolled background included II and III/IV

†The patients were classified into III if their enrolled background included III and IV

### Patient demographics, clinical characteristics, and bacterial pathogens

The demographic and clinical characteristics of the patients with HCAP are provided in Tables 2 and 3. There are no significant differences for gender and age between survivors and non-survivors at 30 days post admission. Patients who smoke have higher all-cause mortality rates than non-smokers.

**Table 2:** Patient demographics characteristics (three-day ICU)

	All *N *= 444	Non-ICU *N *= 333	ICU *N *= 111	*P-value*	Survivors *N *= 351	Non-survivors *N *= 93	*P-value*
Demographics							
- Smoking	191 (43.0)	142 (42.6)	49 (44.1)	0.782	135 (38.5)	56 (60.2)	<0.001
- Male	326 (73.6)	243 (73.2)	83 (74.8)	0.743	252 (72.0)	74 (79.6)	0.141
- Age, yrs	72.1 (15.1)	72 (15.6)	72.5 (13.6)	0.736	71.7 (15.3)	73.7 (14.1)	0.291
- Age ≥ 65 yrs	332 (74.8)	242 (72.7)	90 (81.1)	0.077	260 (74.1)	72 (77.4)	0.509
- Age ≥ 75 yrs	235 (52.9)	171 (51.4)	64 (57.7)	0.249	182 (51.9)	53 (57.0)	0.377
Comorbidity							
- Charlson comorbidity score	2 (1 to 3)	2 (1 to 2)	2 (1 to 3)	0.013	2 (1 to 2)	2 (2 to 3)	<0.001
- Neoplastic disease	166 (37.4)	131 (39.3)	35 (31.5)	0.141	108 (30.8)	58 (62.4)	<0.001
- Liver disease	28 (6.3)	16 (4.8)	12 (10.8)	0.024	21 (6.0)	7 (7.5)	0.586
- Cardiovascular disease	68 (15.3)	43 (12.9)	25 (22.5)	0.015	52 (14.8)	16 (17.2)	0.569
- Cerebrovascular disorders	120 (27.0)	81 (24.3)	39 (35.1)	0.026	100 (28.5)	20 (21.5)	0.177
- CNS	67 (15.1)	56 (16.8)	11 (9.9)	0.078	57 (16.2)	10 (10.8)	0.189
- Renal disease	81 (18.2)	51 (15.3)	30 (27.0)	0.006	67 (19.1)	14 (15.1)	0.370
- Pulmonary disease	114 (25.7)	82 (24.6)	32 (28.8)	0.380	88 (25.1)	26 (28.0)	0.571
- Diabetes mellitus	130 (29.3)	89 (26.7)	41 (36.9)	0.041	103 (29.3)	27 (29.0)	0.953
- Immunocompromised status	54 (12.2)	38 (11.4)	16 (14.4)	0.402	43 (12.3)	11 (11.8)	0.912

*Data are expressed as number count (percentage) or median (interquartile range)

**Table 3:** Patient clinical characteristics (three-day ICU)

	All *N *= 444	Non-ICU *N *= 333	ICU *N *= 111	*P-value*	Survivors *N *= 351	Non-survivors *N *= 93	*P-value*
Clinical features							
- Received ventilation	139 (31.3)	45 (13.5)	94 (84.7)	<0.001	87 (24.8)	52 (55.9)	<0.001
- Septic shock	104 (23.4)	49 (14.7)	55 (49.5)	<0.001	61 (17.4)	43 (46.2)	<0.001
- Altered mental status	111 (25.0)	53 (15.9)	58 (52.3)	<0.001	66 (18.8)	45 (48.4)	<0.001
- Pleural effusion	144 (32.4)	97 (29.1)	47 (42.3)	0.010	101 (28.8)	43 (46.2)	0.001
- Multilobar involvement	242 (54.5)	161 (48.3)	81 (73.0)	<0.001	174 (49.6)	68 (73.1)	<0.001
- Temperature <35°C or ≥40°C	8 (1.8)	3 (0.9)	5 (4.5)	0.026	3 (0.9)	5 (5.4)	0.012
- BUN >20 mg/dL	279 (62.8)	191 (57.4)	88 (79.3)	<0.001	206 (58.7)	73 (78.5)	<0.001
- BUN >30 mg/dL	164 (36.9)	107 (32.1)	57 (51.4)	<0.001	113 (32.2)	51 (54.8)	<0.001
- Pulse ≥125/minute	97 (21.8)	63 (18.9)	34 (30.6)	0.010	81 (23.1)	16 (17.2)	0.223
- Respiratory rate >30/minute	31 (7.0)	14 (4.2)	17 (15.3)	<0.001	24 (6.8)	7 (7.5)	0.817
- Systolic BP <90 mmHg	35 (7.9)	18 (5.4)	17 (15.3)	0.001	21 (6.0)	14 (15.1)	0.004
- Distolic BP ≤60 mmHg	121 (27.3)	84 (25.2)	37 (33.3)	0.097	87 (24.8)	34 (36.6)	0.023
- Haematocrit <30%	144 (32.4)	110 (33.0)	34 (30.6)	0.640	105 (29.9)	39 (41.9)	0.028
- Arterial PH <7.35	65 (14.6)	23 (6.9)	44 (39.6)	<0.001	41 (11.7)	24 (25.8)	0.001
- Glucose ≥250 mg/dL	44 (9.9)	28 (8.4)	16 (14.4)	0.067	34 (9.7)	10 (10.8)	0.760
- PaO2 <60 mmHg	86 (19.4)	49 (14.7)	37 (33.3)	<0.001	59 (16.8)	27 (29.0)	0.005
Initial antibiotic therapy§				0.583			0.001
- Inadequate	75 (16.9)	57 (17.1)	18 (16.2)		52 (14.8)	23 (24.7)	
- Adequate	158 (35.6)	162 (48.6)	49 (44.1)		117 (33.3)	41 (44.1)	
- Indeterminate	211 (47.5)	114 (34.2)	44 (39.6)		182 (51.9)	29 (31.2)	
Outcome							
- Length of ICU stay, days	8 (4 to 17)	------	8 (4 to 17)	---	12 (6.5 to 8.5)	8 (2 to 15.3)	0.002
- Length of hospital stay, days	15 (9 to 25)	15 (8 to 23)	19 (9 to 37)	0.038	17 (9 to 29)	9 (4 to 20)	<0.001
- In-hospital mortality	117 (26.4)	64 (19.2)	53 (47.8)	<0.001	24 (6.9)	93 (100.0)	<0.001

*Data are expressed as number count (percentage) or median (interquartile range)

§Inadequate initial antibiotic therapy was defined as the condition when the therapy was unable to cover any of the isolated bacterium

Neoplasm disease is the most important co-morbidity which causes higher mortality. Other co-morbidities--cerebrovascular disorders, renal disease, liver disease, and diabetes mellitus--can predict a higher need for ICU admission at Day 3.

Many of the predictors that were checked within two days were associated with higher all-cause mortality and the need for ICU admission. The predictors include a patient's requirement for mechanical ventilation, septic shock status, altered mental status, presence of pleural effusion, pneumonia with multilobar involvement, high fever or hypothermia, high BUN level, arterial blood acidosis, and hypoxemia.

The pathogen yielded in patients who were admitted to the ICU at 3 days and at 14 days tended to be Gram negative bacteria. Initial antibiotic choice is crucial and inadequate antibiotic administration could cause higher mortality. *Pseudomonas aeruginosa *was the most frequently found pathogen, followed by *Klebsiella *spp. (Table 4).

**Table 4:** Etiology of healthcare-associated pneumonia

	All	3-day ICU Admission	14-day ICU Admission	30-day Mortality
	*N *= 259*	*N *= 84†	*N *= 91¶	*N *= 58§
**Gram-negative pathogens**				
- *Pseudomonas *spp.	83 (32.0)	25 (29.8)	26 (28.6)	19 (32.8)
- *Klebsiella *spp.	72 (27.8)	23 (27.4)	23 (25.3)	13 (22.4)
- *Acinetobater *spp.	8 (3.1)	2 (2.4)	3 (3.3)	4 (6.9)
- *Escherichia coli*	14 (5.4)	4 (4.8)	6 (6.6)	3 (5.2)
- *Enterbacterium *spp.	14 (5.4)	6 (7.1)	7 (7.7)	4 (6.9)
- *Haemophilus influenzae*	6 (2.3)	4 (4.8)	4 (4.4)	
- *Proteus mirabilis*	6 (2.3)	1 (1.2)	2 (2.2)	1 (1.7)
- *Serratia marcescens*	6 (2.3)	2 (2.4)	2 (2.2)	2 (3.4)
- *Stenotrophmonas maltophilia*	5 (1.9)	2 (2.4)	2 (2.2)	1 (1.7)
- Other	1 (0.3)			
**Gram-positive pathogens**				
- *Streptococcus pneumoniae*	8 (3.1)	5 (6.0)	5 (5.5)	1 (1.7)
- MRSA	22 (8.5)	5 (6.0)	6 (6.6)	7 (12.1)
- MSSA	8 (3.1)	3 (3.6)	3 (3.3)	3 (5.2)
- Other *Streptococcus *spp.	4 (1.5)	2 (2.4)	2 (2.2)	
- Other	1 (0.3)			
**Other**	1 (0.3)			

*From 204 subjects. †From 66 subjects. ¶From 72 subjects. §From 45 subjects.

MRSA: methicillin-resistant *Staphylococcus aureus*

MSSA: methicillin-sensitive *Staphylococcus aureus*

### Scoring indices to predict mortality and ICU admission hospital LOS

As shown in Table 5, the scoring indices originally designed for CAP were tested to be applied to HCAP. The adverse outcome rate increased steadily from low to high, meeting the criteria for all scores. The average LOS increased steadily from low to high, either for risk class or meeting criteria. PSI can offer moderate discriminating ability for separating patients between survivors and non-survivors at 30 days, as well as for predicting the need for ICU admission. The performance of each index in predicting 3-day and 14-day ICU admission and 30-day mortality were also determined (Tables 6 and 7). PSI (>90) has the highest sensitivity to predicting mortality (AUC: 0.70), followed by CURB-65 (≥2) (AUC: 0.66), and SCAP (>9) (AUC: 0.71). For predicting ICU admission (Day 3 and Day 14), modified ATS (AUC: 0.84, 0.82), SMART-COP (AUC: 0.84, 0.82), SCAP (AUC: 0.82, 0.80) and IDSA/ATS (AUC: 0.80, 0.79) performed better (statistically significant difference) than PSI, CURB-65, SOAR and SMRT-CO.

**Table 5:** ICU admission, mortality, and hospital LOS according to different prediction rules

	Patients	3-day ICU Admission	14-day ICU Admission	30-day Mortality	Hospital LOS, d*
**Total number of patients**	444	111	129	93	
**Modified ATS**					
- Low (not meeting criteria)	248 (55.9)	6 (2.4)	13 (5.2)	25 (10.1)	14 (8.3 to 22.8)
- High (meeting criteria)	196 (44.1)	105 (53.6)	116 (59.2)	68 (34.7)	18 (9 to 29.8)
*P-value*		<0.001	<0.001	<0.001	0.013
**IDSA/ATS**					
- Low (not meeting criteria)	234 (52.7)	8 (3.4)	15 (6.4)	22 (9.4)	14 (8.8 to 23)
- High (meeting criteria)	210 (47.3)	103 (49.0)	114 (54.3)	71 (33.8)	17 (9 to 29)
*P-value*		<0.001	<0.001	<0.001	0.058
**SOAR**					
- Low (not meeting criteria)	317 (71.4)	42 (13.2)	56 (17.7)	54 (17.0)	15 (8 to 23)
- High (meeting criteria)	127 (28.6)	69 (54.3)	73 (57.5)	39 (30.7)	17 (9 to 34)
*P-value*		<0.001	<0.001	0.001	0.018
**SCAP**					
- Low (0 to approximately 9)	184 (41.4)	12 (6.5)	17 (9.2)	18 (9.8)	14 (8 to 23)
- Intermediated (10 to approximately 19)	164 (36.9)	41 (25.0)	50 (30.5)	33 (20.1)	16 (9 to 25)
- High (≥20)	96 (21.6)	58 (60.4)	62 (64.6)	42 (43.8)	18 (9 to 34.8)
*P-value*		<0.001	<0.001	<0.001	0.049
**SMART-COP**					
- Low (0 to approximately 2)	275 (61.9)	21 (7.6)	31 (11.3)	35 (12.7)	14 (9 to 23)
- Intermediate (3 to approximately 4)	93 (20.9)	39 (41.9)	43 (46.2)	28 (30.1)	17 (8 to 27)
- High (≥5)	76 (17.1)	51 (67.1)	55 (72.4)	30 (39.5)	17.5 (9 to 32)
*P-value*		<0.001	<0.001	<0.001	0.138
**SMRT-CO**					
- Low (0 to approximately 1)	291 (65.5)	41 (14.1)	51 (17.5)	44 (15.1)	15 (9 to 23)
- Intermediate (2)	83 (18.7)	25 (30.1)	31 (37.3)	22 (26.5)	18 (8 to 29)
- High (≥3)	70 (15.8)	45 (64.3)	47 (67.1)	27 (38.6)	17 (7.8 to 27)
*P-value*		<0.001	<0.001	<0.001	0.431
**CURB65**					
- Low (0 to approximately 1)	142 (32.0)	12 (8.5)	16 (11.3)	12 (8.5)	14 (8 to 23)
- Intermediate (2)	153 (34.5)	33 (21.6)	42 (27.5)	34 (22.2)	15 (9 to 23.5)
- High (≥3)	149 (33.6)	66 (44.3)	71 (47.7)	47 (31.5)	17 (8 to 29)
*P-value*		<0.001	<0.001	<0.001	0.166
**PSI**					
- Low (≤90, Class I to approximately III)	80 (18.0)	8 (10.0)	10 (12.5)	7 (8.8)	12 (7.3 to 20.8)
- Intermediate (91 to 130, Class IV)	205 (46.2)	36 (17.6)	46 (22.4)	33 (16.1)	16 (9 to 24)
- High (>130, Class V)	159 (35.8)	67 (42.1)	73 (45.9)	53 (33.3)	17 (8 to 29)
*P-value*		<0.001	<0.001	<0.001	0.028

*Data are presented as median (interquartile range). Non-parametric Mann-Whitney U test or Jonckheere-Terpstra's trend test was used to examine the statistically significant differences between groups.

**Table 6:** Measure of performance predicting 3-day and 14-day ICU admission and 30-day mortality by using different prediction rules

	Sensitivity	Specificity	PPV	NPV	AUC
**Modified ATS**					
- ICU admission (3 d)	94.6 (88.6 to 98.0)	72.7 (67.5 to 77.4)	53.6 (46.3 to 60.7)	97.6 (94.8 to 99.1)	0.836 (0.799 to 0.870)
- ICU admission (14 d)	89.9 (83.4 to 94.5)	74.6 (69.4 to 79.3)	59.2 (52.0 to 66.1)	94.8 (91.2 to 97.2)	0.823 (0.784 to 0.857)
- Mortality	73.1 (62.9 to 81.8)	63.5 (58.3 to 68.6)	34.7 (28.1 to 41.8)	89.9 (85.5 to 93.4)	0.683 (0.638 to 0.726)
**IDSA/ATS**					
- ICU admission (3 d)	92.8 (86.3 to 96.8)	67.9 (62.6 to 72.9)	49.0 (42.1 to 56.0)	96.6 (93.4 to 98.5)	0.803 (0.763 to 0.839)
- ICU admission (14 d)	88.4 (81.5 to 93.3)	69.5 (64.1 to 74.6)	54.3 (47.3 to 61.2)	93.6 (89.6 to 96.4)	0.789 (0.749 to 0.826)
- Mortality	76.3 (66.4 to 84.5)	60.4 (55.1 to 65.6)	33.8 (27.4 to 40.6)	90.6 (86.1 to 94.0)	0.684 (0.638 to 0.727)
**SOAR**					
- ICU admission (3 d)	62.2 (52.5 to 71.2)	82.6 (78.1 to 86.5)	54.3 (45.3 to 63.2)	86.8 (82.5 to 90.3)	0.724 (0.680 to 0.765)
- ICU admission (14 d)	56.6 (47.6 to 65.3)	82.9 (78.2 to 86.9)	57.5 (48.4 to 66.2)	82.3 (77.7 to 86.4)	0.697 (0.652 to 0.740)
- Mortality	41.9 (31.8 to 52.6)	74.9 (70.1 to 79.4)	30.7 (22.8 to 39.5)	83.0 (78.4 to 86.9)	0.584 (0.537 to 0.631)
**SCAP (>9)**					
- ICU admission (3 d)	89.2 (81.9 to 94.3)	51.7 (46.1 to 57.1)	38.1 (32.1 to 44.3)	93.5 (88.9 to 96.6)	0.818 (0.778 to 0.852)
- ICU admission (14 d)	86.8 (79.7 to 92.1)	53.0 (47.3 to 58.6)	43.1 (37.0 to 49.3)	90.8 (85.6 to 94.5)	0.801 (0.760 to 0.837)
- Mortality	80.7 (71.1 to 88.1)	47.3 (42.0 to 52.7)	28.8 (23.4 to 34.8)	90.2 (85.0 to 94.1)	0.709 (0.664 to 0.751)
**SMART-COP (>2)**					
- ICU admission (3 d)	81.1 (72.5 to 87.9)	76.3 (71.3 to 80.7)	53.3 (45.4 to 61.0)	92.4 (88.6 to 95.2)	0.836 (0.798 to 0.869)
- ICU admission (14 d)	76.0 (67.7 to 83.0)	77.5 (72.4 to 82.0)	58.0 (50.2 to 65.5)	88.7 (84.4 to 92.2)	0.822 (0.783 to 0.857)
- Mortality	62.4 (51.7 to 72.2)	68.4 (63.2 to 73.2)	34.3 (27.2 to 42.0)	87.3 (82.7 to 91.0)	0.686 (0.641 to 0.729)
**SMRT-CO (>1)**					
- ICU admission (3 d)	63.1 (53.4 to 72.0)	75.1 (70.1 to 79.6)	45.8 (37.7 to 54.0)	85.9 (81.4 to 89.7)	0.756 (0.713 to 0.795)
- ICU admission (14 d)	60.5 (51.5 to 69.0)	76.2 (71.1 to 80.8)	51.0 (42.8 to 59.1)	82.5 (77.6 to 86.7)	0.751 (0.708 to 0.791)
- Mortality	52.7 (42.1 to 63.1)	70.4 (65.3 to 75.1)	32.0 (24.7 to 40.0)	84.9 (80.2 to 88.8)	0.672 (0.627 to 0.716)
**CURB-65 (>1)**					
- ICU admission (3 d)	89.2 (81.9 to 94.3)	39.0 (33.8 to 44.5)	32.8 (27.5 to 38.4)	91.5 (85.7 to 95.6)	0.732 (0.688 to 0.772)
- ICU admission (14 d)	87.6 (80.6 to 92.7)	40.0 (34.5 to 45.6)	37.4 (31.9 to 43.1)	88.7 (82.3 to 93.4)	0.715 (0.670 to 0.756)
- Mortality	87.1 (78.5 to 93.1)	37.0 (32.0 to 42.3)	26.8 (21.9 to 32.2)	91.5 (85.7 to 95.6)	0.662 (0.616 to 0.706)
**PSI (>90)**					
- ICU admission (3 d)	92.8 (86.3 to 96.8)	21.6 (17.3 to 26.4)	28.3 (23.7 to 33.2)	90.0 (81.2 to 95.6)	0.730 (0.868 to 0.771)
- ICU admission (14 d)	92.3 (86.2 to 96.2)	22.2 (17.8 to 27.2)	32.7 (27.9 to 37.8)	87.5 (78.2 to 93.8)	0.717 (0.673 to 0.759)
- Mortality	92.5 (85.1 to 96.9)	20.8 (16.7 to 25.4)	23.6 (19.4 to 28.3)	91.3 (82.8 to 96.4)	0.703 (0.658 to 0.745)

Data are presented as percentages (95% confidence interval)

The scores were dichotomized as low risk vs. higher risk (Modified ATS: meeting criteria, IDSA/ATS: meeting criteria, SOAR: meeting criteria, SCAP >9, SMART-COP >2, SMRT-CO >1, CURB-65 >1, PSI >90).

**Table 7:** Pairwise comparison of ROC curves (the number represents the p-value)

	Modified ATS	IDSA/ATS	SOAR	SCAP	SMART-COP	SMRT-CO	CURB-65	PSI
Modified ATS		***0.984***	†, ***0.006***	***0.443***	***0.934***	***0.769***	***0.588***	***0.623***
IDSA/ATS	0.070/0.066		†, ***0.008***	***0.458***	***0.948***	***0.750***	***0.561***	***0.627***
SOAR	#, 0.001/<0.001	#, 0.024/0.005		†, <***0.001***	†, ***0.001***	†, ***0.013***	†, ***0.028***	†, ***0.002***
SCAP	0.532/0.436	0.640/0.697	#, 0.001/<0.001		***0.309***	***0.215***	***0.152***	***0.836***
SMART-COP	0.996/0.985	0.286/0.259	#, <0.001/<0.001	0.358/0.259		***0.555***	***0.526***	***0.647***
SMRT-CO	#, 0.015/0.020	0.146/0.209	0.339/0.086	#, 0.020/0.049	#, <0.001/<0.001		***0.777***	***0.456***
CURB-65	#, 0.003/0.001	#, 0.034/0.018	0.807/0.577	#, 0.003/0.001	#, 0.001/<0.001	0.461/0.240		***0.223***
PSI	#, 0.003/0.001	#, 0.037/0.028	0.854/0.548	#, 0.001/0.001	#, 0.001/<0.001	0.477/0.316	0.960/0.930	

*The cells in bold and italics represent the p-value in pairwise comparison for predicting the 30-day mortality, the normal cells represent the *P*-value for predicting the ICU-admission (3-day/14-day)

† Statistically significant difference in predicting 30-day mortality

# Statistically significant difference in predicting both 3-day and 14-day ICU admission.

## Discussion

HCAP is a heterogeneous disease that includes patient populations with varying severities of illness [22]. The mortality associated with HCAP was similar to that of nosocomial pneumonia, higher than that of CAP, and lower than ventilator-associated pneumonia [13]. As shown in Table 1, each subgroup contributes to different parts of overall HCAP mortality. There is increased mortality of groups II (34.4%) and III (47.3%) of patients with HCAP, indicating that HCAP is a heterogeneous disease. As has already been reported by Brito and Niederman, all patients with HCAP should be identified and then divided on the basis of severity of illness to guide initial therapy [13]. Severe pneumonia has been defined by the requirement for admission to an ICU [16]. The decision to admit a patient with HCAP to an ICU depends on subjective clinical views and the peculiarities of the local healthcare setting. The availability of valid criteria for defining severe pneumonia would provide a more reliable basis for improving patient risk assessments. The severity on admission can affect hospital mortality, the need for ICU admission, and even 90-day mortality after hospital discharge [23]. A number of prognostic scoring tools have been developed to predict mortality and the need for ICU care for patients with CAP; the two tools that have been studied the most are the PSI and CURB-65. However, they are not ideal for assessing the need for ICU care, and other scoring systems, such as those developed by the IDSA/ATS guideline group, and the SMART-COP tool, are available for this purpose [24]. So far, and to the best of our knowledge, no severity index has been developed and validated for patients with HCAP.

The AUC is a measure of the accuracy of a test to correctly classify patients with and without a particular outcome and is used frequently in studies of severity assessment in CAP. The AUC describes the relationships between sensitivity and specificity, a higher AUC implies a less steep trade-off between sensitivity and specificity. An AUC is considered to have moderate discriminating power from a value of 0.70 on up. We conducted this retrospective chart review of 444 records and assessed the validity of PSI, CURB-65, SCAP, and others and constructed an ROC.

The PSI scoring system has been shown to be a powerful tool for assigning the risk of death from CAP in different populations [17]. This scoring system was primarily designed to identify patients with a low mortality risk who could safely be treated as outpatients. However, it is complicated to use, requiring computation of a score based on 20 variables. To ensure that the final prediction rule remained simple to use and practical, prognostic features not usually available at the time of initial assessment post hospital admission were excluded from the CURB-65 model [21]. The CURB-65 model does not consider decompensated co-morbidity due to CAP and results in limited application in the elderly [24]. Since the majority of patients were elderly, the data are not much different from what is published in the literature regarding CAP; that is, CURB-65 may not be a good index for predicting mortality in this population.

The modified ATS rule provides simple clinical criteria for those patients who require ICU admission [16]. According to the authors' description, the modified ATS rule can serve as a useful counterpart to the prediction by Fine *et al. *The modified ATS rule was good in terms of sensitivity (89.9%) and the area under the receiver operator curve graph (0.823) for predicting 14-day ICU admission in HCAP patients. The modified ATS severe CAP definition published in 2001 was superseded by the 2007 IDSA-ATS severe CAP definition (IDSA/ATS). The newer definition was based on a series of papers and on re-evaluation by the guideline committee of data published since the 2001 definition was made. Therefore, we also tested the two indices and found that modified ATS as well as IDSA/ATS can be applied for defining severe HCAP.

The strongest clinical predictors of SCAP were pH <7.30 and systolic pressure <90 mmHg [19]. A depressed pH, which is likely a side effect of metabolic acidosis derived from sepsis, is not included in other prediction rules, such as CURB-65 or modified ATS. In our series, a low pH was associated with poor outcomes in patients with HCAP. The SCAP score is as accurate as, or better than, other current scoring systems (for example, CURB-65 and PSI) in predicting adverse outcomes in patients hospitalized with CAP [25]. We found that SCAP also works well with HCAP. The discriminatory power of SCAP, as measured by AUC, was 0.81 for ICU admission in our HCAP patients, compared with the 0.75 in CAP patients from another study [25].

The PSI and CURB-65 have been used to guide the need for ICU care, but they are not ideal for this purpose [24]. Some of these indices were originally designed to assess ICU admission rather than mortality. Therefore, a poor performance could be found if applied in predicting mortality. Compared to PSI, modified ATS, IDSA/ATS, SCAP, and SMART-COP were easy to calculate. For predicting ICU admission (Day 3 and Day 14), modified ATS (AUC: 0.84, 0.82), SMART-COP (AUC: 0.84, 0.82), SCAP (AUC: 0.82, 0.80) and IDSA/ATS (AUC: 0.80, 0.79) performed better (showing a statistically significant difference) than PSI, CURB-65, SOAR and SMRT-CO.

The main strength of the study is the relatively large sample size. The limitations of the study include possible selection bias as all patients who were included in our analysis consist of a heterogenic variety of sources. There may be different patient characteristics in each study site. On the other hand, it can reflect the reality of HCAP coming from heterogeneous populations. In addition, there are a huge number of patients that received microbiologically adequate therapy (sensitive to the antibiotic administered) and their clinical conditions do not improve because of other possible factors (for example, incorrect dosing, interval of administration, pharmacokinetic/pharmacodynamic features, hypoalbuminemia in critically ill patients) which were not investigated in this study. However, those were beyond the scope of the study.

## Conclusions

The utility of the scoring indices for risk assessment in patients with healthcare-associated pneumonia shows that the scoring indices originally designed for CAP can be applied to HCAP. The promising results offer the clinician an adjunctive tool when making site-of-treatment decisions for patients and when stratifying patients with HCAP into risk groups.

## Key messages

• There is currently no scoring index to predict the outcomes of patients with HCAP, a type of pneumonia that occurs prior to hospital admission in patients with specific risk factors following contact or exposure to a healthcare environment.

• We applied and compared different community acquired pneumonia (CAP) scoring indices to predict 30-day mortality and 3-day and 14-day intensive care unit (ICU) admission in patients with HCAP.

• PSI has the highest sensitivity in predicting mortality, followed by CURB-65 (≥2) and SCAP (>9) (SCAP score (AUC: 0.71), PSI (AUC: 0.70) and CURB-65 (AUC: 0.66)).

• For predicting ICU admission (Day 3 and Day 14), modified ATS (AUC: 0.84, 0.82), SMART-COP (AUC: 0.84, 0.82), SCAP (AUC: 0.82, 0.80) and IDSA/ATS (AUC: 0.80, 0.79) performed better (statistically significant difference) than PSI, CURB-65, SOAR and SMRT-CO.

• The promising results offer the clinician an adjunctive tool when making site-of-treatment decisions for patients and when stratifying patients with HCAP into risk groups.

## Abbreviations

AUC: area under the curve; BAL: bronchoalveolar lavage; CAP: community acquired pneumonia; CURB 65: confusion, urea, respiratory rate, blood pressure, age 65; HCAP: healthcare-associated pneumonia; IDSA/ATS: Infectious Diseases Society of America/American Thoracic Society; LOS: length of hospital stay; NPV: negative predictive value; PPV: positive predictive value; PSB: protected sheath brushing; PSI: pneumonia severity index; ROC: receiver operating characteristic; SCAP: severe community acquired pneumonia; SMART-COP: systolic blood pressure, multilobar involvement, albumin, respiratory rate, tachycardia, confusion, oxygenation, pH; SMRT-CO: systolic blood pressure, multilobar involvement, respiratory rate, tachycardia, confusion, oxygenation; SOAR: systolic blood pressure, oxygenation, age, respiratory rate.

## Competing interests

The authors declare that they have no competing interests.

## Authors' contributions

FWF carried out study design, analysis and interpretation of data, and drafted the manuscript. YKY, CJW, CJY, CWC, CYT, and MCL were principal investigators of each study medical center, participating in the study design and coordination, and helped to draft the manuscript. All authors read and approved the final manuscript.
